# Trends of Modern Contraceptive Use among Young Married Women Based on the 2000, 2005, and 2011 Ethiopian Demographic and Health Surveys: A Multivariate Decomposition Analysis

**DOI:** 10.1371/journal.pone.0116525

**Published:** 2015-01-30

**Authors:** Abebaw Gebeyehu Worku, Gizachew Assefa Tessema, Atinkut Alamirrew Zeleke

**Affiliations:** 1 Department of Reproductive Health, Institute of Public Health, University of Gondar, Gondar, Ethiopia; 2 Department of Health Informatics, Institute of Public Health, University of Gondar, Gondar, Ethiopia; Kuopio University Hospital, FINLAND

## Abstract

**Introduction:**

Accessing family planning can reduce a significant proportion of maternal, infant, and childhood deaths. In Ethiopia, use of modern contraceptive methods is low but it is increasing. This study aimed to analyze the trends and determinants of changes in modern contraceptive use over time among young married women in Ethiopia.

**Methods:**

The study used data from the three Demographic Health Surveys conducted in Ethiopia, in 2000, 2005, and 2011. Young married women age 15–24 years with sample sizes of 2,157 in 2000, 1,904 in 2005, and 2,146 in 2011 were included. Logit-based decomposition analysis technique was used for analysis of factors contributing to the recent changes. STATA 12 was employed for data management and analyses. All calculations presented in this paper were weighted for the sampling probabilities and non-response. Complex sampling procedures were also considered during testing of statistical significance.

**Results:**

Among young married women, modern contraceptive prevalence increased from 6% in 2000 to 16% in 2005 and to 36% in 2011. The decomposition analysis indicated that 34% of the overall change in modern contraceptive use was due to difference in women’s characteristics. Changes in the composition of young women’s characteristics according to age, educational status, religion, couple concordance on family size, and fertility preference were the major sources of this increase. Two-thirds of the increase in modern contraceptive use was due to difference in coefficients. Most importantly, the increase was due to change in contraceptive use behavior among the rural population (33%) and among Orthodox Christians (16%) and Protestants (4%).

**Conclusions:**

Modern contraceptive use among young married women has showed a remarkable increase over the last decade in Ethiopia. Programmatic interventions targeting poor, younger (adolescent), illiterate, and Muslim women would help to maintain the increasing trend in modern contraceptive use.

## Introduction

Modern family planning service in Ethiopia pioneered by Family Guidance Association (FGAE) established in 1966 [1]. After 1980, the Ministry of Health expanded its family planning (FP) services with cyclic country support programs by UNFPA and other stakeholders. With the adoption of the population policy in 1993 [2], numerous local and international partners in family planning have partnered with the government in expanding FP programs and services. In 1996, the Ministry of Health released Guidelines for Family Planning Services in Ethiopia to guide health providers and managers, as well as to expand and ensure quality family planning services in the country [1]. The Government of Ethiopia and Non-Governmental Organizations (NGOs) Expand community-based distribution of family planning services at the women’s door level through health extension program since 2002. The government has removed all duty and taxes on imported contraceptives since family planning is considered as key for the country’s development [3].

Ethiopia is one of three countries (along with Malawi and Rwanda) in sub-Saharan Africa that have achieved a much more rapid increase in the contraceptive prevalence rate (CPR) than any other country in the region in the last 10 years, according to an analysis of modern contraceptive prevalence in recent Demographic and Health Surveys (DHS) [3]. In Ethiopia the change for contraceptive among married women aged 15–49 was from 8% in 2000 to 29% in 2011[4,5] which is still low but is steadily increasing. The type of modern contraception is almost the same across the years, while the proportion of each contraceptive type is different, in which injectable and implant are increasing while there is a significant decrease of pill over the years.

Young women, especially adolescents, are at higher risk of morbidity and mortality associated with pregnancy and childbirth [6]. The risk is much higher when pregnancy is unintended while most pregnancies to young girls in sub-Saharan Africa are unintended or mistimed [7]. In Ethiopia 28% of adolescents aged 15–19 and 24% of young women aged 20–24 have had unintended pregnancies [5].

Family planning is a key investment in reducing the broader costs of health care [8] and reducing risks associated with pregnancy and childbirth. Accessing family planning can reduce maternal deaths by 40% [9,10], infant mortality by 10%, and childhood mortality by 21% [9]. In Ethiopia every dollar invested in family planning has shown two dollars of savings in other development areas [11,12]. It is well known that reproductive choices made by young women and men have an enormous impact on their prospects for health, schooling, and employment, as well as their overall transition to adulthood [13–15].

Studies in Ethiopia indicate that women in the richest household wealth quintile, educated women, employed women, urban women, and women who follow the Christian religion tend to use modern contraception more than other women [16–18]. Moreover, having a higher number of living children [19], being in a monogamous relationship, having family size concordance (couple’s agreement on family size), desire to limit or space births, attending community conversation, and being visited by health workers at home have been identified as determinants of modern contraceptive use [16,18].

Identifying the contributing factors to the changes in contraceptive use among women age 15–24 helps to improve young women’s contraceptive use. The increasing trend in contraceptive use could be due to the current changes in population composition, including urbanization, education of girls, and other development activities, or it could be due to changes in contraceptive behavior. Researchers have conducted decomposition analyses to understand and explain the sources of changes in the use of contraception in recent decades. A study in Cameroon showed that between 1991 and 2004 more than 37% of the change in contraceptive use among women aged 15–49 years was due to coefficients. However, compositional changes contributed more (45%) to the increase in modern contraceptive use. Improvements in husbands’ positive attitudes toward contraceptive use, secondary school education, and couple’s discussion on contraceptive use contributed the most to increases in contraceptive use [20].

In a study in Rwanda, about three-quarters (78%) of the change in modern contraceptive use between 2005 and 2010 among women aged 15–49 years was due to changes in effects of women’s characteristics, particularly women’s education and place of residence [21]. A study in Ethiopia using decomposition analysis showed that 69% of the change in contraceptive use during the period 2000–2011 was attributable to compositional changes, and about 23% of the overall contraceptive increase was attributable to changes in coefficients. The change was mainly due to an increase in the effects of religion, experience of child death, ideal family size, and working for pay among women [18].

Therefore, investigating the major factors contributing to such a dramatic change helps to plan strategies for family planning programs. Hence, this research answers the following questions

1. What are the levels and trends in contraceptive use among young married women age 15–24 in Ethiopia over a 10-year period?
2. What are the main factors contributing to the recent changes in contraceptive use among young married women in Ethiopia?

## Methods

### Data

**Figure 1:**
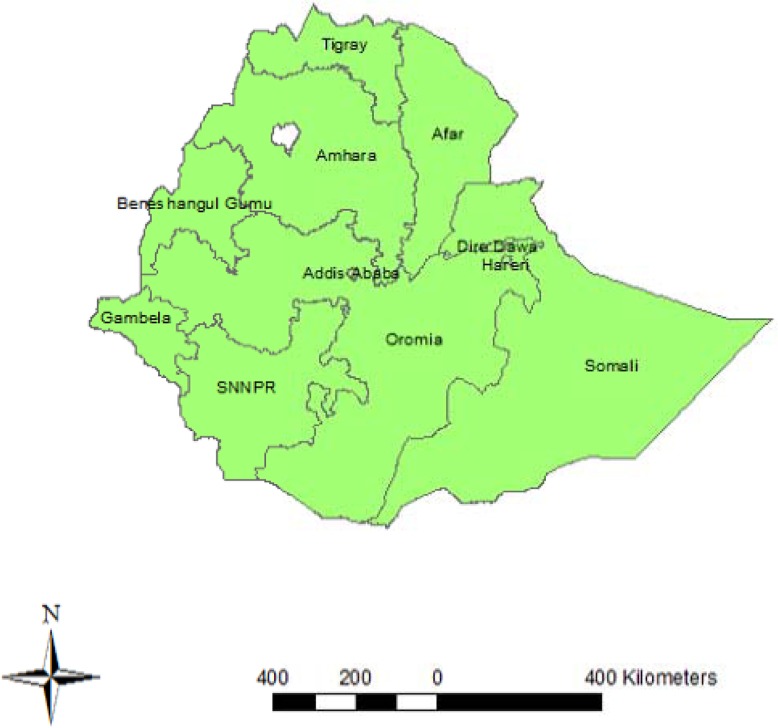
Regional States of Federal Democratic Republic of Ethiopia.

The data for this study were accessed from the DHS program official database. The DHS collects data through nationally representative cross-sectional surveys in over 40 developing countries. The survey is usually conducted at five-year intervals in a country. Ethiopia has undertaken three consecutive DHS surveys, in 2000, 2005, and 2011. The Ethiopian DHS was planned to have estimates according to the 11 regional states (9 regions and 2 city administrations) (Fig. 1)

**Figure 2:**
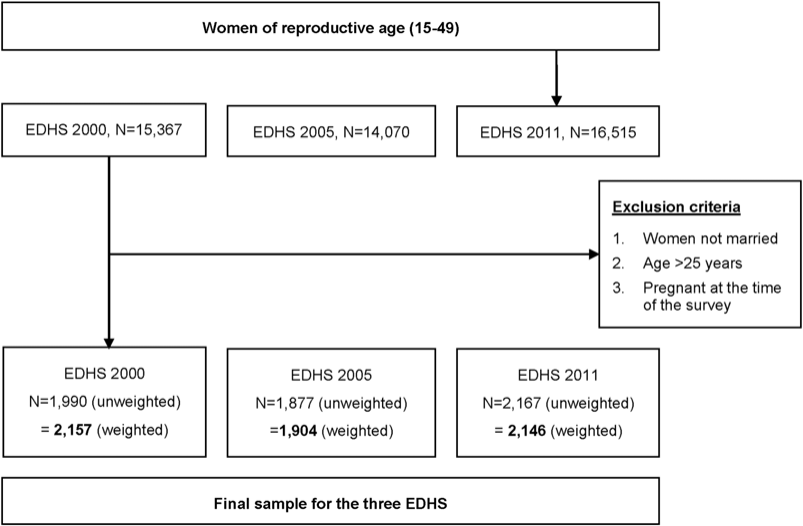
The exclusion procedures to identify the final sample size in DHS 2000, 2005, and 2011.

In this study, our data are restricted to married and non-pregnant women aged 15–24. Based on these criteria, our sample sizes from the three Ethiopian Demographic and Health Surveys (EDHS) were 1,990 women in 2000 (2157 weighted cases), 1,877 in 2005 (1904 weighted cases), and 2,167 in 2011(2146 weighted cases) (Fig. 2)).

### Key Variables and Measurements

The study variables were classified into dependent and independent variables. The dependent variable was current modern contraceptive use, categorized dichotomously as a “Yes/No” variable. Respondents who were currently using a modern contraceptive method were categorized as “Yes”, otherwise as “No”. In this study, modern contraceptive methods include female and male sterilization, oral contraceptive pill, Intra-uterine device (IUD) injectables, implants, and condom.

The key independent variables were the following:

**Socio-demographic variables**. Age [15–19, 20–24], residence [rural, urban], region (9 regions and 2 administrative areas), religion [Orthodox, Muslim, Protestant, Others], wealth index [poorest, poorer, middle, richer, richest], women’s education [no education, primary, secondary and above], partner’s education [no education, primary, secondary and above], working status [not working, working but not paid/paid in kind, paid in cash], and number of living children [0,1, 2, 3+].

**Fertility preference and decision-making**. Family planning size concordance [both want the same, husband wants more, husband wants fewer, do not know/missing]; women’s participation in decision-making [not participated, participated]; and fertility preference [wants soon, wants later, wants no more].

**Family planning program exposure**. For the study, being visited by family planning workers in the last 12 months was dichotomized as “Yes” and “No”. Similarly, knowing about different contraceptive methods is likely to have a positive effect on modern contraceptive use. Thus in the study being knowledgeable about family planning was classified as “Yes” for knowledgeable and “No” for non-knowledgeable.

### Statistical Analysis

This study employed trend analysis of modern contraceptive use and decomposition of changes in modern contraceptive use. The trend in modern contraceptive use was analyzed using descriptive analyses, stratified by region, urban-rural residence, and selected socio-demographic characteristics. The trend was examined separately for the periods 2000–2005, 2005–2011, and 2000–2011.

Multivariate decomposition analysis of change in modern contraceptive use was employed to answer the major research question of this study. The analysis was a regression decomposition of the difference in modern contraceptive use between two surveys (the 2000 and 2011 EDHS data). The purpose of the decomposition analysis was to identify the sources of changes in the use of modern contraception in the last decade. Both changes in population composition and population behavior related to contraceptive use (effect) are important. This method is used for several purposes in demography, economics, and other fields. The present analysis focused on how use of contraception responds to changes in women’s characteristics and how these factors shape differences across surveys conducted at different times. The technique utilizes the output from logistic regression model to parcel out the observed difference in contraceptive use in to components. This difference can be attributed to compositional changes between surveys (i.e. differences in characteristics) and to changes in effects of the selected explanatory variables (i.e. differences in the coefficients due to changes in population behavior). Hence, the observed difference in modern contraceptive use between different surveys is additively decomposed into a characteristics (or endowments) component and a coefficient (or effects of characteristics) component. STATA 12 was employed for data management and analyses. STATA commands were applied during the process of analysis. All calculations presented in this paper were weighted for the sampling probabilities and non-response using the weighting factor included in the EDHS data. During testing of statistical significance or associations (95% confidence interval calculations), complex sampling procedures were considered. The process was done by using the SVY STATA command to control the clustering effect of complex sampling (stratification and multistage sampling procedures).

### Ethical considerations

Ethiopian DHS obtained ethical clearance from Ethiopian Health Nutrition and Research Institute (EHNRI) Review Board, the National Research Ethics Review Committee (NRERC) at the Ministry of Science and Technology of Ethiopia, the Institutional Review Board (IRB) of ICF International, and the Center for Disease Control (CDC). During the data collection, the interviewer read aloud a statement to get consent from the respondents. The respondents provided verbal consent, as DHS is conducted in areas where not all respondents are able to write. The interviewers then signed their name to document that the statement was read and that consent was granted or declined. Children were not respondents to interview; however, parent/guardians gave consent for measurements. Detailed information on the methodology and ethical issue was published in the Ethiopian Demographic and Health Survey reports [4,5,22].

The authors have submitted proposal to DHS Program/ICF International and permission was granted to download and use the data for this study. The DHS Program authorized data access; and data were used solely for the purpose of the current study.

## Results

### Characteristics of the Study Population

Tables 1 and 2 presents the characteristics of respondents (married women aged 15–24) over the three EDHS periods. Among the respondents, about seven out of ten in all three surveys were aged 20–24. In terms of residence, in 2000 and 2005, 10% of respondents resided in urban areas, increasing to 18% in 2011. With regard to educational status, in the first two surveys about three-quarters (79% in 2000 and 74% in 2005) were not educated, while in EDHS 2011 only 49% were not educated. The proportion with primary education rose from 15% in 2000 to 41% in 2011. Across the three DHS surveys, the proportion of Orthodox Christians showed a slight decline, from 52% to 47% between 2000 and 2011, while Protestants increased from 14% to 20% (Table 1).

**Table 1:** Percentage distribution of socio-demographic characteristics of the respondents, 2000, 2005, and 2011 Ethiopia Demographic and Health Surveys.

**Characteristics**		**2000**	**2005**	**2011**
		**N = 2,157**	**N = 1,904**	**N = 2,146**
**Age**	15–19 years	31.5	30.7	30.0
	20–24 years	68.5	69.3	70.0
**Residence**	Urban	10.2	10.1	17.8
	Rural	89.8	89.9	82.2
**Region**	Tigray	8.1	6.7	7.3
	Afar	1.4	1.4	1.2
	Amhara	29.6	30.6	30.6
	Oromiya	40.5	37.1	39.7
	Somali	0.8	3.2	1.9
	Benishangul-Gumuz	1.2	1.3	1.3
	SNNPR	16.4	16.5	14.0
	Gambela	0.4	0.4	0.6
	Harari	0.2	0.3	0.3
	Addis Ababa	1.2	2.0	2.8
	Dire Dawa	0.2	0.4	0.3
**Religion**	Orthodox	51.5	49.0	46.8
	Muslim	31.5	32.6	31.5
	Protestant	13.6	15.6	19.5
	Other/missing	3.5	2.8	2.2
**Educational status**	None	79.1	73.5	49.2
	Primary	14.6	19.2	40.6
	Secondary or above	6.3	7.4	10.2
**Partner’s educational status**	None	56.2	51.9	39.9
	Primary	31.3	33.3	44.8
	Secondary or above	12.5	14.8	15.3
**Working status**	Not working	39.1	71.9	48.7
	Working but not paid/paid in kind only	43.8	21.5	22.8
	paid with cash	17.1	6.6	28.5
**Wealth index**	Poorest	9.8	22.8	26.4
	Poorer	18.6	18.4	16.8
	Middle	26.8	17.9	16.1
	Richer	21.4	15.6	15.4
	Richest	23.5	25.3	25.4

**Table 2:** Percentage distribution of fertility preference and family planning program exposure of the respondents, 2000, 2005, and 2011 Ethiopia Demographic and Health Surveys.

**Characteristics**		**2000**	**2005**	**2011**
		**N = 2,157**	**N = 1,904**	**N = 2,146**
**FERTILITY PREFERENCE AND DECISION-MAKING**				
**Number of living children**	0	25.1	22.3	25.5
	1	38.7	39.4	37.6
	2	24.0	25.5	26.3
	3+	12.2	12.8	10.6
	Mean	1.1	1.2	1.2
**Family size concordance**	Both want same	35.3	37.0	47.9
	Husband wants more	21.0	14.3	18.6
	Husband wants fewer	3.6	4.2	5.9
	Do not know/missing	40.2	44.5	27.6
**Fertility Preference**	Wants soon	30.3	22.1	22.1
	Wants later	56.7	58.2	64.2
	Wants no more	10.8	18.8	11.6
	Undecided	2.2	0.9	2.2
**Women’s decision-making**	Not participated	Na	57.4	47.8
	Participated	Na	42.6	52.2
**FAMILY PLANNING PROGRAM EXPOSURE**				
**Knowledge of modern contraceptives**	No	16.7	13.0	2.8
	Yes	83.3	87.0	97.2
**Visited by health worker**	No	98.5	93.2	87.2
	Yes	1.5	6.8	12.8
**FP media exposure**	No	83.7	72.9	66.3
	Yes	16.3	27.1	33.7

The mean number of living children remained more or less similar across all three surveys. Concerning family size concordance, the percentage of husbands who wanted more children than their wives declined from 23% in 2000 to 14% in 2005, rising again to 19% in 2011. The percentage of husbands wanting the same number of children as their wives wanted (concordance) rose from 35% in 2000 to 48% in 2011 (Table 2).

Regarding young women’s fertility preference, no difference was observed between 2000 and 2011 in the proportion wanting no more children. In all three surveys only a small proportion of women reported having been visited by a health worker, but the percentage rose from less than 2 percent in 2000 to 13% in 2011. The proportion of women with exposure to family planning information in the media doubled, from 16% in 2000 to 34% in 2011 (Table 2).

Except for age, region, religion, and number of living children, all other variables listed in Table 1 and 2 showed significant changes, when comparing the sample population in year 2000 with 2011.

### Trends in Modern Contraceptive Use

**Figure 3:**
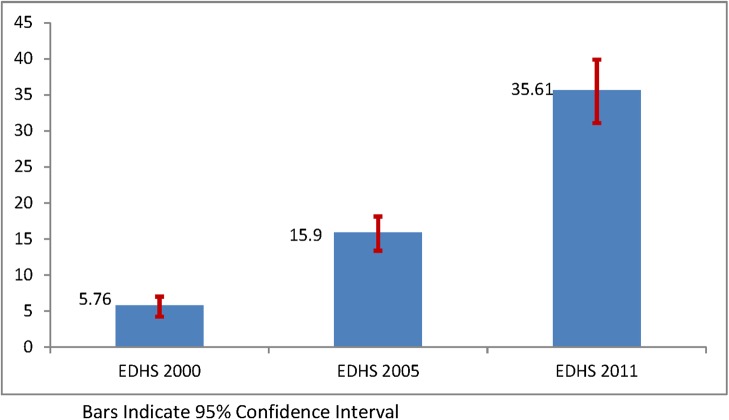
Trends in contraceptive use among Ethiopian young married women in the past 10 years, Ethiopia Demographic and Health Surveys 2000–2011.

This section presents trends in contraceptive use during the period 2000–2011. Looking at the overall trend, Ethiopia has shown a significant increase in modern contraceptive use among young married women over the study period, from 6% in 2000 to 16% in 2005, and to 36% in 2011 (Fig. 3).

The trend period was divided in to two phases, 2000–2005 and 2005–2011, to see the differences in increase over time. The largest increase in modern contraceptive use was seen in the second phase (2005–2011) with a 19.7 percentage point change compared with a 10.4 percentage point change in the first phase (2000–2005) (Table 3).

**Table 3:** Trend in modern contraceptive use among young currently married women by selected characteristics, 2000, 2005, and 2011 Ethiopia Demographic and Health Surveys.

**Characteristics**	**EDHS 2000 n = 2,157**	**EDHS 2005 n = 1,904**	**EDHS 2011 n = 2,146**	**Percentage point difference in modern contraceptive use**		
				**Phase I 2005–2000**	**Phase II 2011–2005**	**Overall 2011–2000**
**Residence**						
Urban	35.0	51.4	66.7	16.4	15.3	31.7
Rural	2.4	11.9	28.9	9.5	16.9	26.4
**Region**						
Tigray	8.7	17.1	24.5	8.4	7.4	15.8
Afar	11.4	11.0	11.4	-0.4	0.4	0.0
Amhara	4.8	17.3	38.5	12.6	21.2	33.8
Oromia	3.9	14.3	33.4	10.4	19.1	29.5
Somali	4.6	0.0	3.8	-4.6	3.8	-0.8
Benishangul-Gumuz	14.1	13.3	41.4	-0.8	28.1	27.3
SNNP	5.8	13.5	37.8	7.7	24.3	32.0
Gambela	12.0	23.6	47.7	11.6	24.0	35.7
Harari	24.1	32.1	39.6	8.1	7.5	15.5
Addis Ababa	48.3	62.1	79.2	13.8	17.2	31.0
Dire Dawa	28.3	35.3	38.4	7.1	3.1	10.2
**Age**						
15–19 years	3.9	10.4	27.3	6.6	16.9	23.4
20–24 years	6.6	18.3	39.2	11.7	20.9	32.6
**Women’s education**						
No education	2.6	10.3	24.4	7.7	14.2	21.9
Primary	10.8	24.7	41.0	13.9	16.3	30.1
Secondary +	34.2	49.5	68.5	15.3	19.0	34.3
**Husbands’ education**						
No education	2.1	9.1	24.5	7.1	15.3	22.4
Primary	6.2	19.2	38.4	13.0	19.2	32.3
Secondary +	21.3	32.4	56.5	11.0	24.1	35.2
**Religion**						
Orthodox	6.6	21.4	42.3	14.8	20.9	35.7
Muslim	5.0	12.1	22.2	7.1	10.1	17.2
Protestant	5.9	8.5	43.7	2.6	35.2	37.8
Others	0.0	5.2	14.4	5.2	9.2	14.3
**Working status**						
Not working	6.3	14.0	34.5	7.7	20.5	28.1
Working but not paid	2.7	15.7	27.2	13.0	11.5	24.5
Paid with cash	12.3	37.4	44.3	25.1	6.9	32.0
**Visited by health worker**						
Yes	(36.9)	20.4	38.6	-16.5	18.2	1.7
No	5.2	15.6	35.2	10.4	19.6	30.0
**FP media exposure**						
No	3.8	10.0	27.6	6.2	17.6	23.8
Yes	15.6	31.8	51.2	16.2	19.4	35.6
**Family size concordance**						
Both want same	9.3	23.9	50.0	14.5	26.1	40.7
Husband wants more	5.8	11.6	26.3	5.8	14.7	20.5
Husband wants fewer	9.1	23.1	39.7	14.0	16.6	30.6
Do not know/missing	2.3	9.9	21.3	7.6	11.4	19.0
Total	5.8	15.9	35.6	10.1	19.7	29.9

Figures are presented in percentages of users from weighted sample (n), Figures in “( )” indicate sample size is <50

The trends in modern contraceptive use by young women showed variation according to their characteristics. Major increases in modern contraceptive use were observed in some of the categories. Among rural residents, the largest increase in modern contraceptive use was observed during the second phase of the study period (2005–2011), at a 16.9 percentage point increase compared with a 9.5 percentage point increase during the first phase (2000–2005).

Three regions—Amhara, Oromia, and SNNPR—showed the greatest increases in modern contraceptive use between 2000 and 2011. Young women with secondary and above education showed a 34.3 percentage point increase in modern contraceptive use between 2000 and 2011.

Both young age groups (15–19 and 20–24) showed an increase in modern contraceptive use. Women aged 20–24 showed a larger increase in contraceptive use than women aged 15–19. Regarding family size concordance, among women whose husbands wanted the same number of children as they did, modern contraceptive use increased by 40.7 percentage points between 2000 and 2011, nearly twice as much as among women whose husbands wanted more children than they did (20.5 percentage points) (Table 3).

### Decomposition Analysis

The decomposition analysis revealed that about 34% of the overall percentage change in modern contraceptive use by young married women was due to difference in characteristics (compositional factors). Among the compositional factors, a significant contribution to the positive change in modern contraceptive use was associated with women’s education. Education is an important variable for use of contraception (Table 4). Thus an increase in the composition of women’s attainment of primary and above education over the survey period (Table 1) showed a significant contribution to the change in modern contraceptive use (Table 4).

**Table 4:** Decomposition of change in modern contraceptive use among young married women in Ethiopia, 2000 to 2011.

**Modern contraceptive use**		**Difference due to characteristics (E)**		**Difference due to coefficients (C)**	
		**Coefficient**	**Percent**	**Coefficient**	**Percent**
Age	15–19 years				
	20–24 years	0.00133**	0.44522	0.01218	4.07930
Residence	Urban				
	Rural	0.01202***	4.02580	0.09849**	32.99700
FP media exposure	No				
	Yes	0.00857	2.86960	0.00056	0.18869
Women’s education	None				
	Primary	0.02170**	7.26820	-0.00042	-0.13943
	Secondary+	0.00723**	2.42230	-0.00114	-0.38330
Husbands’ education	None				
	Primary	0.00210	0.70214	-0.01365	-4.57230
	Secondary+	-0.00100	-0.33537	-0.00382	-1.27900
Religion	Muslim				
	Orthodox	-0.00770**	-2.57820	0.04824**	16.16200
	Protestant	0.00879**	2.94380	0.01295*	4.33810
	Others/Missing	0.00034	0.11506	0.01268***	4.24880
Occupation	Not working				
	Paid in kind	0.00991	3.31950	-0.01263	-4.23260
	Paid with cash	0.00254	0.84942	-0.00641	-2.14660
Family size concordance	Both want same				
	Husband wants more	0.00356**	1.19130	-0.00803	-2.68980
	Husband wants fewer	-0.00135	-0.45294	0.00097	0.32619
	Don’t know/ Missing	0.02186**	7.32320	-0.00319	-1.06850
Number of living children	0				
	1	0.00002	0.00626	0.00856	2.86700
	2	-0.00033	-0.11104	-0.00046	-0.15287
	3+	0.00124	0.41442	0.00038	0.12588
Fertility preference	Wants soon				
	Wants later	0.00917**	3.07120	-0.00198	-0.66481
	Wants no more	0.00099*	0.33123	-0.00591	-1.98040
	Undecided	0.00003	0.00902	0.00308	1.03020
Constant				0.05662	18.97000
Total		0.10140**	33.97700	0.197**	66.02300

*Significant at 0.05

**Significant at 0.01

***Significant at <0.001

Although compositional changes were small (Table 1), an increase in the proportion of young women aged 20–24 in the sample made a significant contribution to increasing use of modern contraception (Table 4). A decreasing proportion of Orthodox Christians in the sample (Table 1) showed a significant negative impact on the use of modern contraceptive methods. On the other hand, an increasing proportion of Protestants in the sample was associated with a significant contribution to the percentage increase of modern contraceptive use (Table 4).

Another compositional factor affecting modern contraceptive use was family size concordance. A decline over the survey period in the proportion of husbands wanting to have more children than their wives wanted significantly contributed to the increasing use of modern contraceptive use in the last decade. Similarly, an increase in the composition of women with fertility preference for spacing and limiting births showed significant contributions to the observed changes in modern contraceptive use among young women (Table 4).

After controlling for the role of compositional changes, 66% of the increase in modern contraceptive use was due to difference in the effects of characteristics. Factors including residence and religion showed a significant effect for the observed positive change in modern contraceptive use. Other things being equal, about one-third of the increase in modern contraceptive use in the past decade was due to a change in modern contraceptive use behavior among the rural population. Compared with followers of the Muslim religion, followers of other religions, especially the Orthodox Christian religion, showed a significant contribution to the observed percentage increase in modern contraceptive use over the decade. The effect of religion has become more important over time (Table 4).

## Discussion

This study was planned to examine the trends and the major factors positively or negatively contributing to the changes in modern contraceptive use in the past 10 years.

Young women’s modern contraceptive use increased substantially over the last decade particularly in the second survey period, 2005–2011. Ethiopia is one of the three counties in sub-Saharan Africa with the most rapid increase in modern contraceptive use [3]. This may be attributed to rigorous family planning programs by the government and NGOs, through improvement in the health care infrastructure and government attention to meeting the MDG goals through health sector development strategies [3].

About a third of the overall change in modern contraceptive use by young married women was due to difference in characteristics. The finding is much lower than the previous reports based on analysis of women age 15–49 in Ethiopia [18] and a study in Cameroon [20]. This implied that a significant contribution of the change arises when the composition of the population changes according to important variables.

An increase in the composition of women’s attainment of primary and above education showed a significant effect on modern contraceptive use. Currently, girls’ education is on the government priority agenda in Ethiopia. Therefore, the proportion of educated young women is expected to rise and to continue having an impact on modern contraceptive use in the future.

Compositional changes by categories of religion were associated with both negative and positive impacts on the trend in use of contraception. A decreasing proportion of Orthodox Christians (who use contraception more than Muslims) negatively affected the trends in modern contraceptive prevalence, and an increasing proportion of Protestants (who also use contraception more than Muslims) had a positive effect on the prevalence trends. The finding implies that appropriate strategies are needed to improve service access and benefits of family planning programs, especially in Muslim-dominated regions of the country.

The decline over time in the proportion of husbands who want more children than their wives want has contributed significantly to increasing modern contraceptive use in the last decade. This finding can reflect an increasing trend of discussion and agreement between husbands and wives on family size and contraceptive use. In this case, the decision of the husband is particularly important in patriarchic societies like Ethiopia.

An increase in the composition of women having a fertility preference for spacing and limiting their births contributed significantly to the observed changes. This can be a reflection of a growing awareness and positive attitude on the importance of family planning. A decline in desire for having more children can reflect the growing engagement of young women in development activities, including education.

About two-thirds of the increase in modern contraceptive use over the past decade was due to difference in the effects of characteristics (coefficients). This finding is in line with a study in Rwanda, where most of the increase in modern contraceptive use was found to be due to change in coefficients [21].

One of the striking findings from the decomposition analysis was the effect of residence. About a third of the increase in modern contraceptive use among young married women in the past decade was due to changes in modern contraceptive use behavior of the rural population. Although urban women’s prevalence of modern contraceptive use (67%) still far exceeds rural prevalence (29%), the biggest increase has been in the rural areas, where most of the population lives (an eleven-fold increase in rural areas compared with a two-fold increase in urban areas). Government commitments to improve family planning and other health service access might explain this finding. As noted in previous studies [3,23], the implementation of the Health Extension Program is the major reason, with provision of family planning services in the rural areas and recently in urban settings of the country. For this purpose, more than 33,000 Health Extension Workers were deployed, mostly in the rural parts of the country. Family planning, one of the 16 packages of rural health care provided by Health Extension Workers, has as its objectives to address misconceptions concerning family planning in the community, to counsel clients on all family planning methods, and to provide short-acting modern contraceptive methods [24].

Another remarkable finding in this part of the analysis was the effect of religion. The changes in modern contraceptive use behavior differ significantly among the categories of religion. Most of the increase in modern contraceptive use since 2000 has been among followers of the Orthodox Christian and Protestant religions. As recognized in previous studies [18], the effect of religion has become more important over time. Although there is no supporting evidence on the reasons for the difference among religions, religious belief is one of the psychosocial barriers when women think about using a method for fertility regulation. However, studies may need to understand the major reasons for slow progress in adopting family planning, in order to identify factors with programmatic relevance.

This study had a number of strengths. First, the study utilized large datasets representing the whole country, and thus the findings were based on adequate statistical power. Second, calculations were done after the data were weighted for the sampling probabilities and non-response. Complex sampling procedures were also considered during testing of statistical significance. Third, analytic techniques such as decomposition analysis were applied, to understand the sources of change in modern contraceptive use.

This study highlighted important findings to support family planning program in Ethiopia, but it is not without limitations, which could affect conclusions based on some of the findings. During decomposition analysis, important variables such as women’s decision-making, representing women’s autonomy, were not included due to lack of data about this variable in the 2000 EDHS. However, some of the contribution of this variable can be represented by the variable “couple’s concordance on family size”. Comparability of the wealth index across the three surveys was difficult, as the EDHS measurement tools for the wealth index were different among the surveys, and hence this variable was excluded from the trend analysis. Due to lack of studies conducted among young women aged 15–25 years, we were forced to consider studies conducted among women aged 15–49 years old.

## Conclusions

Modern contraceptive use among young married women has shown a remarkable increase over the last decade in Ethiopia. One-third of the overall change in modern contraceptive use by young married women over the decade was due to difference in characteristics between the 2000 and 2011 EDHS. Changes in the composition of young women’s characteristics according to age, educational status, religion, couple concordance on family size, and fertility preference were the major sources of the increase in modern contraceptive prevalence among this group.

Two-thirds of the increase in modern contraceptive use was due to change in contraceptive use behavior. Most importantly, the increase was due to change in contraceptive use behavior among the rural population and among Orthodox Christians and Protestants.

Program interventions, including health behavior education and family planning services and counseling, are especially needed for some categories of the population, including poor, younger (adolescents), illiterate, and Muslim young married women. Strengthening community-based and school-based family planning programs are strategies to maintain young women’s contraceptive use and to advance it further. It is mandatory to continue education of the young population, as education is one of the major factors contributing to increasing contraceptive use among young people in Ethiopia.
